# Prospective validation of multimodal risk stratification using clinical and quantitative imaging features for 12-month outcomes after minimally invasive surgery for lumbar disc herniation

**DOI:** 10.3389/frai.2026.1909448

**Published:** 2026-10-16

**Authors:** Jichong Zhu, Hao Li, Chunxian Lu, Hengkua Huang, Tianyou Chen, Sitan Feng, Jiarui Chen, Ruifei Bai, Xinli Zhan, Chong Liu, Chengqian Huang

**Affiliations:** 1 The First Affiliated Hospital of Guangxi Medical University, Nanning, China; 2 The People's Hospital of Baise City, Baise, China; 3 The Second Affiliated Hospital of Guangxi Medical University, Nanning, China; 4 Spinal Orthopedic Surgery, Affiliated Hospital of Youjiang Medical University for Nationalities, Baise, Guangxi, China; 5 Liuzhou Railway Central Hospital, Liuzhou, China; 6 Key Laboratory of Molecular Pathology in Tumors of Guangxi Higher Education Institutions, Baise, Guangxi, China

**Keywords:** lumbar disc herniation, minimally invasive surgery, multimodal fusion, prognostic prediction model, prospective study

## Abstract

**Background:**

Lumbar disc herniation (LDH) is a common spinal disorder leading to chronic low back pain and radiating leg pain. Minimally invasive surgery is the preferred treatment, but postoperative recovery varies significantly, making accurate preoperative prognosis prediction a challenge.

**Methods:**

This study developed and prospectively validated a multimodal risk-stratification framework integrating structured clinical variables, conventional imaging parameters, CT/MRI radiomics, and high-dimensional image-derived features. A total of 773 patients were analyzed, including 665 patients in the retrospective development cohort and 108 patients in an independent prospective validation cohort. The retrospective cohort was divided into a training cohort (*n* = 532) and an internal validation cohort (*n* = 133). Feature selection, parameter optimization, and classification-threshold determination were performed exclusively within the training cohort, and the finalized framework was subsequently evaluated in the internal and prospective validation cohorts.

**Results:**

The integrated multimodal approach achieved an ROC-AUC of 0.937 (95% CI, 0.900–0.975) and a PR-AUC of 0.849 in the internal validation cohort, and an ROC-AUC of 0.976 (95% CI, 0.951–1.000) and a PR-AUC of 0.941 in the prospective validation cohort. Its discrimination was comparable to that achieved using structured clinical and conventional imaging information alone and was higher than that of isolated quantitative imaging approaches. At the prespecified training-derived classification threshold of 0.318, sensitivity, specificity, and balanced accuracy in the prospective cohort were 85.3%, 95.9%, and 90.6%, respectively. Calibration analysis demonstrated residual probability miscalibration despite strong discrimination, although training-derived logistic recalibration improved the Brier score.

**Conclusion:**

Multimodal integration of clinical and quantitative imaging information demonstrated strong discrimination for 12-month postoperative outcome stratification in both internal and prospective validation cohorts. However, the incremental discriminative value beyond structured clinical and conventional imaging information was limited, and residual miscalibration remained in the prospective cohort. Further multicenter validation, standardized quantitative imaging workflows, and calibration assessment are warranted before clinical translation.

## 1 Introduction

Lumbar disc herniation (LDH) is a common spinal disorder, primarily characterized by chronic low back pain and radiating leg pain, significantly impacting patients' quality of life (GBD 2021 Low Back Pain Collaborators, 2023). With the continuous advancement of minimally invasive techniques, minimally invasive surgery has become the preferred treatment option for LDH. However, postoperative recovery remains heterogeneous despite technically successful surgery (Foster et al., 2018), as patient-related factors, preoperative symptoms, and structural characteristics may influence long-term pain and functional outcomes. Therefore, accurate preoperative risk stratification remains an important clinical challenge (Liu et al., 2025).

Previous studies have identified several clinical and imaging factors associated with postoperative outcomes after lumbar disc surgery, including demographic characteristics, baseline symptoms, and degenerative imaging findings. However, these conventional predictors often provide limited individualized prognostic information because postoperative recovery is influenced by multiple interacting biological and structural factors. Recently, quantitative imaging approaches, including radiomics and deep-learning-based feature extraction (Lambin et al., 2012), have enabled the characterization of imaging information beyond conventional visual assessment (Zwanenburg et al., 2020). Similarly, multiparametric MRI texture analysis has demonstrated that quantitative imaging signatures can capture predictive information beyond conventional visual assessment in other clinical settings (Nong et al., 2024). When combined with clinical variables, multimodal prediction frameworks may provide complementary information for postoperative risk stratification (Kelly et al., 2019; Xuan et al., 2023).

Therefore, this study aimed to develop and prospectively validate a multimodal prognostic model integrating structured clinical variables, conventional imaging parameters, radiomics features, and deep-learning-derived features for predicting 12-month postoperative symptom states after minimally invasive surgery for LDH. We further compared the multimodal model with isolated imaging-based models and a structured clinical/conventional-imaging model to evaluate the incremental value of quantitative imaging information. By integrating multiple preoperative information domains, this study aimed to evaluate whether quantitative imaging representations could provide additional prognostic information beyond structured clinical and conventional imaging variables. The findings may contribute to a more comprehensive understanding of individualized postoperative risk stratification after minimally invasive surgery for LDH.

## 2 Methods

### 2.1 Study design and cohort selection

This study consisted of a retrospective model-development cohort and an independent prospective validation cohort. Similar prospective observational designs have also been applied in clinical prediction studies from regional spine and orthopedic cohorts. The retrospective cohort included 665 patients with lumbar disc herniation (LDH) who underwent minimally invasive surgery and had complete baseline clinical, laboratory, imaging, and 12-month follow-up data. The retrospective cohort was subsequently divided by stratified random sampling into a training cohort (*n* = 532) and an internal validation cohort (*n* = 133), as described in Section 2.5.

Patients were consecutively enrolled according to predefined inclusion and exclusion criteria. The retrospective cohort was used for model development, including feature selection, model training, and internal validation. After completion of model development, an independent prospective cohort was consecutively recruited and followed for 12 months to evaluate the locked model. Only patients with complete 12-month clinical outcome assessments were included in the final prospective validation analysis.

The eligibility criteria for the prospective cohort were prespecified to be consistent with those used for the retrospective cohort to improve comparability between populations. No prospective patient data were used during feature selection, hyperparameter optimization, model fitting, classification-threshold determination, or recalibration-model fitting. The overall study design and downstream leakage-control strategy are illustrated in Figure 1.

**Figure 1:**
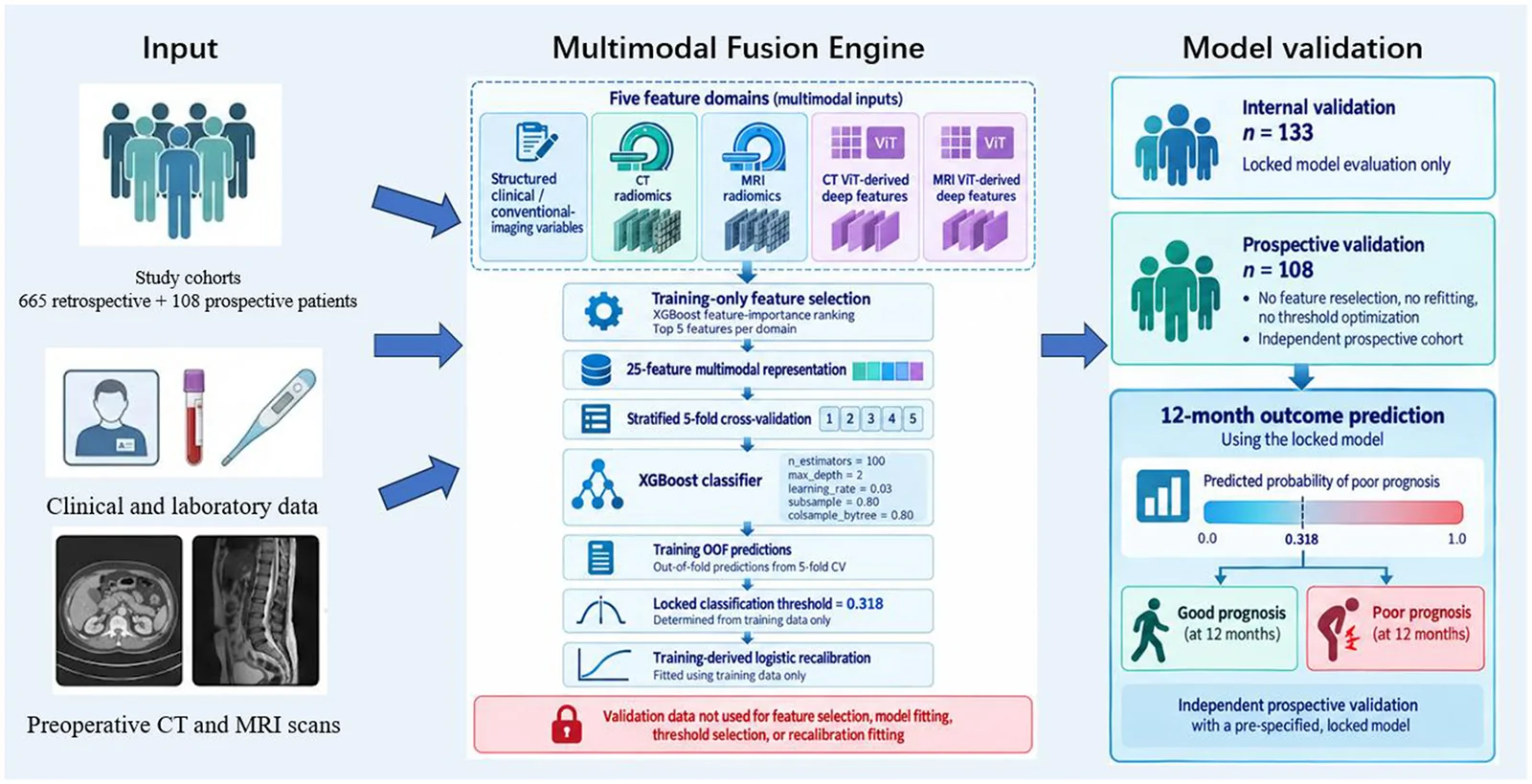
Study design and training-only downstream multimodal modeling workflow. The study included a retrospective model-development cohort of 665 patients and an independent prospective validation cohort of 108 patients. Five feature domains were analyzed, including structured clinical/conventional-imaging variables, CT radiomics, MRI radiomics, CT ViT-derived deep features, and MRI ViT-derived deep features. Feature selection, hyperparameter optimization, out-of-fold prediction generation, classification-threshold determination, and logistic recalibration were performed using training data only. The final locked multimodal model was then evaluated in the internal validation cohort and the independent prospective validation cohort for 12-month outcome prediction after minimally invasive surgery for lumbar disc herniation.

### 2.2 Data collection and preprocessing

Clinical data include the patients' demographic information and medical history. Demographic information includes age, gender, weight, height, and body mass index (BMI). The medical history records the presence of conditions such as hypertension, diabetes mellitus, smoking history, alcohol consumption, and family history (e.g., whether family members have a history of cardiovascular disease, cancer, etc.). In addition, preoperative assessments include the evaluation of pain intensity using the Visual Analog Scale (VAS) and the assessment of functional status using the Oswestry Disability Index (ODI).

Laboratory test results and imaging data were included in this study's data collection. Laboratory data consisted of routine hematological tests, liver function (such as ALT, AST), and renal function (such as creatinine and urea nitrogen) markers. Imaging data included preoperative X-ray, CT, and MRI scans of the patients. Various imaging measurements were taken. The foraminal height in the affected segment was determined by measuring the distance between the midpoint of the inferior edge of the pedicle and the midpoint of the superior edge of the pedicle (Supplementary Figure S1A). The Pfirrmann grading of MRI images was used to classify intervertebral disc degeneration into five grades: Grade I (normal disc), Grade II (mild degeneration), Grade III (moderate degeneration), Grade IV (severe degeneration), and Grade V (very severe degeneration) (Pfirrmann et al., 2001). Facet orientation (FO) and facet tropism (FT) were assessed by measuring the angles between the facet joint midline and the facet joint lines (Supplementary Figure S1B). Here, line AB passes through the center of the lumbar vertebral body and the midpoint of the spinous process, while lines CD and EF are the facet joint lines between the peaks of the superior facet. The facet joint angle is the angle between the facet joint midline and the facet joint line. FO is calculated as (αR + αL)/2, and FT is calculated as | αR – αL | (Kalichman et al., 2009). The lumbar lordosis angle (LLA) and sacral slope (SS) were calculated by measuring the angle between the endplate of L1 and the endplate of S1 for LLA, and the angle between the endplate of S1 and the horizontal line for SS (Supplementary Figure S1C) (Patel et al., 2025). The disc height index (DHI) was calculated by the formula DHI = (*a*+*b*) / (*c*+*d*), where “*a*” is the anterior disc height, “*b*” is the posterior disc height, “*c*” is the superior disc width, and “*d*” is the inferior disc width (Supplementary Figure S1D) (Akeda et al., 2015). Additionally, the patients' treatment history (such as prior spinal surgery or non-surgical treatments) and postoperative follow-up data (including complications and recovery status) were also collected and used for postoperative functional recovery analysis.

Imaging data were obtained from preoperative CT and MRI scans. ROI segmentation and image cropping were performed for radiomics analysis. Previously generated deep-learning feature matrices were used for the deep-learning domains, as described in Section 2.3.2. No missing values were present in the finalized clinical, radiomics, or deep-learning feature matrices used for model development and validation; therefore, no statistical imputation was required.

The primary clinical outcome was the 12-month postoperative symptom state. A state-based definition was prespecified to characterize residual pain and disability at the end of follow-up. Good prognosis, corresponding to a minimal symptom state, was defined as both a VAS score ≤ 2 and an ODI score ≤ 20% at 12 months, whereas poor prognosis was defined as failure to achieve this state (VAS >2 or ODI >20%). These thresholds were selected to reflect low residual pain and minimal functional disability and are consistent with previously reported minimal-symptom-state criteria after lumbar spine surgery (Crawford et al., 2019). This absolute postoperative-state definition was chosen instead of a change-based minimal clinically important difference (MCID) because the primary objective was to identify patients with clinically relevant residual symptoms at 12 months rather than to quantify the magnitude of improvement from baseline.

### 2.3 Imaging feature extraction

#### 2.3.1 Radiomics feature extraction

Radiomics analysis was performed separately on preoperative axial CT soft-tissue images and axial T2-weighted MR images using the PyRadiomics framework (van Griethuysen et al., 2017). A two-dimensional radiomics workflow was adopted (force2D = True). Regions of interest (ROIs) were manually and independently annotated by two senior spine surgeons according to a standardized annotation protocol. The two readers independently performed ROI segmentation according to the same predefined protocol. The two readers independently performed ROI segmentation without access to the other reader's annotations. Rectangular ROIs were placed over the target region on the selected axial image and stored as ROI annotation files. The annotated region was subsequently cropped according to its bounding box and padded to a square field of view using a black background. The cropped image was converted to grayscale, and a corresponding binary mask was generated, with the ROI assigned a label value of 255.

Gray-level discretization was performed using a fixed bin width of 20. Radiomic features were extracted from the original image and from Laplacian-of-Gaussian (LoG), local binary pattern (LBP2D), and wavelet-filtered images. For LoG filtering, sigma values of 1, 2, and 3 were used. The extracted feature classes included 2D shape, first-order statistics, gray-level co-occurrence matrix (GLCM), gray-level dependence matrix (GLDM), gray-level run-length matrix (GLRLM), gray-level size-zone matrix (GLSZM), and neighboring gray-tone difference matrix (NGTDM) features. A total of 660 radiomic features were obtained separately from CT and MRI, yielding 1,320 initial radiomic features before modality-specific feature selection.

The same radiomics extraction settings were applied to the retrospective training cohort, internal validation cohort, and prospective validation cohort (Zwanenburg et al., 2020). No explicit voxel-spacing resampling, intensity normalization, or scanner-specific harmonization procedure was applied during radiomics feature extraction. Feature definitions followed those implemented in PyRadiomics; no separate phantom-based IBSI compliance verification was performed.

For inter-reader reproducibility analysis, the 108 patients in the prospective validation cohort were independently annotated by two senior spine surgeons using the same predefined ROI segmentation protocol. Spatial agreement between readers was quantified using the Dice similarity coefficient (DSC) and intersection-over-union (IoU). In addition, inter-reader reliability of ROI width, height, and area was assessed using a two-way random-effects, absolute-agreement, single-measure intraclass correlation coefficient [ICC(A,1)]. Ninety-five percent confidence intervals for ICC(A,1) were estimated using nonparametric bootstrap resampling with 10,000 replicates and a fixed random seed of 42. Repeated segmentation by the same reader was not available; therefore, intra-reader reproducibility was not quantified. Detailed results are presented in Supplementary Table S1.

#### 2.3.2 Deep-learning feature extraction

Deep-learning features were available separately for preoperative axial CT soft-tissue images and axial T2-weighted MR images. The archived feature tables contained one 768-dimensional feature vector per patient for each imaging modality. In the retained data-processing workflow, the feature tables themselves did not contain outcome labels; outcome labels were subsequently linked by patient identifier for downstream supervised modeling.

Available records described the upstream image encoder as a Vision Transformer-Base (ViT-Base) architecture. However, the original ViT implementation and model configuration were not retained. Consequently, the pretrained-weight source, specific feature-output layer, exact network-specific input resizing and normalization procedures, and whether fine-tuning had been performed could not be independently verified from the available records. The upstream feature-generation process therefore could not be fully reconstructed or independently audited.

The fixed pre-generated CT and MRI feature matrices were treated as input variables for the current downstream modeling analysis. Feature selection was performed separately for the CT and MRI deep-learning domains using the training-only procedure described in Section 2.4. Five features from each domain were retained for the final multimodal model. Neither the internal validation cohort nor the prospective validation cohort was used for downstream supervised feature selection or XGBoost model fitting.

### 2.4 Multimodal feature selection and fusion

Radiomic feature extraction was performed using prespecified, outcome-independent settings, whereas all supervised downstream procedures, including feature ranking, feature selection, normalization, scaling, and model fitting, were restricted to the corresponding training data.

To reduce dimensionality while preserving modality-specific information, feature selection was performed separately within each of the five feature domains. During cross-validation, XGBoost-based feature-importance ranking was fitted exclusively within each training fold, and the five highest-ranking features from each domain were selected for that fold. The XGBoost feature selector used 50 estimators, a maximum tree depth of one, a learning rate of 0.05, a subsampling ratio of 0.80, a column-sampling ratio of 0.80, and a fixed random seed of 42.

After completion of the cross-validation procedure, feature selection was refitted using the entire training cohort, and the five highest-ranking features from each domain were retained to construct the final model. All downstream preprocessing procedures used in the current modeling analysis, including feature normalization, scaling, and any parameter estimation required for feature transformation, were performed exclusively within the corresponding training data and subsequently applied unchanged to held-out data. No principal component analysis (PCA) was used in the final modeling pipeline.

Neither the internal validation cohort nor the prospective validation cohort contributed to feature ranking, feature selection, or model fitting. For the pre-generated deep-learning feature matrices, the leakage-control procedures described above apply to the downstream supervised modeling pipeline; the historical upstream ViT feature-generation process could not be independently audited because the original implementation and training records were unavailable.

### 2.5 Model development and internal validation

The retrospective cohort of 665 patients was divided using stratified random sampling into a training cohort (80%, *n* = 532) and an internal validation cohort (20%, *n* = 133), while preserving the outcome distribution between the two cohorts. A fixed random seed of 42 was used for data splitting. Model development was performed exclusively within the training cohort. During five-fold cross-validation, all model training and optimization procedures were repeated within each training fold, while the corresponding validation fold was used only for performance evaluation.

Stratified five-fold cross-validation was used during model development. Within each cross-validation iteration, all supervised feature-selection procedures were fitted exclusively using the corresponding training folds and subsequently applied to the held-out fold. Thus, no information from the held-out fold, internal validation cohort, or prospective validation cohort was used during feature selection, hyperparameter optimization, or model fitting.

Hyperparameter optimization of the XGBoost classifier was performed exclusively within the training cohort using stratified five-fold cross-validation, with ROC-AUC as the optimization criterion. The predefined search space included 50, 100, or 200 estimators, a maximum tree depth of one or two, and a learning rate of 0.03 or 0.10. The subsampling ratio and column-sampling ratio were fixed at 0.80, and the random seed was set to 42. For the final multimodal model, the optimal hyperparameters were 100 estimators, a maximum tree depth of two, and a learning rate of 0.03. The corresponding out-of-fold ROC-AUC in the training cohort was 0.887.

After completion of model development, the complete feature-selection and XGBoost modeling pipeline was refitted using all 532 patients in the training cohort. The resulting model was subsequently locked and applied without further feature selection, hyperparameter optimization, or model fitting to the internal validation cohort and the prospective validation cohort. All data-driven procedures, including feature selection, preprocessing parameter estimation, model training, and hyperparameter optimization, were performed exclusively using the training cohort. The validation cohorts were used only for independent performance evaluation. No oversampling, undersampling, synthetic resampling, or class weighting was applied to the final XGBoost model. Outcome distributions were preserved using stratified data splitting and stratified five-fold cross-validation, and performance under class imbalance was additionally evaluated using PR-AUC and balanced accuracy. All statistical analyses and machine-learning procedures were implemented in Python using scikit-learn, XGBoost, SHAP, and PyRadiomics. The exact package versions used in the original execution environment were not retained and therefore could not be retrospectively verified.

### 2.6 Model evaluation and classification threshold

Poor prognosis was defined as the positive class (label = 1), whereas good prognosis was defined as the negative class (label = 0) for all model-development and evaluation procedures. Model discrimination was assessed using the area under the receiver operating characteristic curve (ROC-AUC) (Collins et al., 2024b). The area under the precision–recall curve (PR-AUC) was additionally calculated because identification of patients with poor prognosis represented the primary clinical objective. Sensitivity, specificity, positive predictive value (PPV), negative predictive value (NPV), F1 score, balanced accuracy, and overall accuracy were also reported. Ninety-five percent confidence intervals (95% CIs) were calculated for model-performance estimates. ROC-AUC confidence intervals were derived using DeLong's method. Wilson score intervals were used for sensitivity, specificity, positive predictive value, negative predictive value, and overall accuracy. Stratified nonparametric bootstrap resampling with 10,000 replicates and a fixed random seed of 42 was used to estimate 95% CIs for PR-AUC, F1 score, balanced accuracy, and Brier score. Calibration intercept and calibration slope were estimated using logistic calibration models, with corresponding 95% Wald confidence intervals. Pairwise comparisons of ROC-AUC between the multimodal model and individual modality models were performed using DeLong's test.

To avoid optimization bias in the validation cohorts, the classification threshold was determined exclusively from out-of-fold predicted probabilities generated by five-fold cross-validation in the training cohort. The threshold maximizing the Youden index was selected and locked before evaluation of the internal and prospective validation cohorts. The resulting training-derived threshold for the final multimodal model was 0.318. No threshold optimization was performed using either validation cohort. Performance at the conventional probability threshold of 0.50 was additionally examined to evaluate the influence of threshold selection on sensitivity and specificity.

Model calibration was evaluated using calibration curves, calibration slope, calibration intercept, and Brier score. As a sensitivity analysis, logistic recalibration was fitted using only out-of-fold predictions from the training cohort and was subsequently applied without further fitting to the internal and prospective validation cohorts. Decision curve analysis (DCA) was performed to quantify clinical net benefit across a range of decision thresholds (Zhao et al., 2024). SHapley Additive exPlanations (SHAP) analysis was used to quantify the contribution of individual features to model predictions (Ponce-Bobadilla et al., 2024).

### 2.7 Prospective validation cohort

A prospective validation cohort was established to independently evaluate the generalizability of the locked multimodal prognostic model. Between April 2024 and February 2025, a total of 120 patients were consecutively recruited. Of these, 108 patients with complete 12-month outcome data were included in the final prospective validation analysis, whereas 12 patients without complete 12-month follow-up data were excluded. The prospective validation protocol received ethical approval from the Ethics Committee of the First Affiliated Hospital of Guangxi Medical University (Approval No. 2025-E0104), and written informed consent was obtained from all prospectively enrolled participants. The final study report incorporating the 108-patient prospective cohort was subsequently reviewed and approved by the same institutional ethics committee (Approval No. 2026-E0050).

The eligibility criteria were prespecified to be consistent with those of the retrospective cohort. Patients were eligible if they had a diagnosis of LDH, underwent minimally invasive nucleus pulposus removal via foraminoscopic surgery, had complete baseline clinical data, and had preoperative CT and MRI images of sufficient quality for analysis. Patients were excluded if they had severely incomplete clinical data, severe cardiovascular, pulmonary, hepatic, renal, or systemic disease, a history of previous spinal surgery or other major concomitant spinal disorders, cognitive or psychiatric conditions preventing reliable follow-up, or pregnancy or breastfeeding.

Demographic characteristics, medical history, clinical symptoms, laboratory tests, and imaging data were collected using the same definitions as those used for the retrospective cohort. Follow-up was completed 12 months after surgery through outpatient evaluation and/or telephone follow-up. Postoperative pain and disability were assessed using the Visual Analog Scale (VAS) and Oswestry Disability Index (ODI), respectively. The same prespecified 12-month outcome definition described in Section 2.2 was applied to the prospective validation cohort.

Before prospective validation, the feature-selection procedure, trained XGBoost model, model hyperparameters, classification threshold, and training-derived recalibration procedure were locked. No model refitting, feature reselection, hyperparameter optimization, or threshold optimization was performed using the prospective cohort.

## 3 Results

### 3.1 Clinical characteristics and conventional predictors

#### 3.1.1 Data analysis of training and validation cohorts

The retrospective model-development cohort comprised 665 patients, including 473 patients with good prognosis and 192 patients with poor prognosis, and was divided into a training cohort (*n* = 532) and an internal validation cohort (*n* = 133). An additional 108 patients with complete 12-month follow-up constituted the independent prospective validation cohort. Baseline characteristics of the retrospective cohort are summarized in Table 1. In terms of gender, there was no significant difference between the two groups (*P* = 0.261). However, age (*P* < 0.001) and BMI (*P* < 0.001) showed significant differences, with patients in the poor prognosis group being older and having a higher BMI.

**Table 1:** Baseline clinical characteristics of patients in the training and internal validation cohorts.

Variable	Type	Good prognosis	Poor prognosis	*P*-value
		(*N* = 473)	(*N* = 192)	
Group	Training	378 (79.9%)	154 (80.2%)	1
	Validation	95 (20.1%)	38 (19.8%)	
Gender	Female	195 (41.2%)	89 (46.4%)	0.261
	Male	278 (58.8%)	103 (53.6%)	
Age	Mean ± SD	49.7 ± 12.3	56.0 ± 13.9	**< 0.001**
BMI	Mean ± SD	23.6 ± 3.0	24.5 ± 3.1	**< 0.001**
Smoking	No	471 (99.6%)	192 (100%)	0.904
	Yes	2 (0.4%)	0 (0%)	
Hypertension	No	436 (92.2%)	156 (81.2%)	**< 0.001**
	Yes	37 (7.8%)	36 (18.8%)	
Diabetes	No	454 (96%)	182 (94.8%)	0.637
	Yes	19 (4%)	10 (5.2%)	
Disease duration	Mean ± SD	31.0 ± 43.8	39.5 ± 59.5	0.072
L3/4	No	451 (95.3%)	183 (95.3%)	1
	Yes	22 (4.7%)	9 (4.7%)	
L4/5	No	188 (39.7%)	73 (38%)	0.745
	Yes	285 (60.3%)	119 (62%)	
L5/S1	No	307 (64.9%)	128 (66.7%)	0.732
	Yes	166 (35.1%)	64 (33.3%)	
Surgical approach	Interlaminar approach	242 (51.2%)	91 (47.4%)	0.427
	Transforaminal approach	231 (48.8%)	101 (52.6%)	
Affected side	Right	204 (43.1%)	86 (44.8%)	0.76
	Left	269 (56.9%)	106 (55.2%)	
Herniation types	Protrusion	3 (0.6%)	1 (0.5%)	0.929
	Herniation	303 (64.1%)	120 (62.5%)	
	Extrusion	156 (33%)	65 (33.9%)	
	Sequestration	11 (2.3%)	6 (3.1%)	
Herniation location	Lateral type	383 (81%)	147 (76.6%)	0.304
	Central type	26 (5.5%)	10 (5.2%)	
	Far lateral type	64 (13.5%)	35 (18.2%)	

Bold values indicate statistical significance (*P* < 0.05).

Additionally, a higher proportion of patients in the poor prognosis group had hypertension (*P* < 0.001), while there were no significant differences in the incidence of diabetes and smoking/alcohol history (*P* = 0.637 and *P* = 0.904, respectively). Regarding imaging data, the involvement of L3/4, L4/5, and L5/S1 in the two groups showed no significant differences (*P* = 1.000, *P* = 0.745 and *P* = 0.732). Similarly, no significant differences were observed in surgical approach and affected side (*P* = 0.427 and *P* = 0.760).

According to the laboratory test results presented in Table 2, significant differences were found in several clinical indicators between the good prognosis and poor prognosis groups. Cystatin C (CysC) (*P* = 0.003) showed a significant difference, with a value of 1.1 ± 0.3 mg/L in the good prognosis group and 1.2 ± 0.4 mg/L in the poor prognosis group, suggesting that CysC may be associated with postoperative recovery. Albumin (ALB) (*P* < 0.001) also showed a significant difference, with a value of 40.9 ± 3.6 g/L in the good prognosis group compared to 39.8 ± 3.6 g/L in the poor prognosis group. Lower ALB levels may be related to poorer postoperative recovery.

**Table 2:** Laboratory test results for good and poor prognosis groups.

Variable	Good prognosis (*N* = 473)	Poor prognosis (*N* = 192)	*P*-value
GGT	43.1 ± 60.1 U/L	43.3 ± 61.8 U/L	0.976
ALT	28.6 ± 25.7 U/L	27.9 ± 26.5 U/L	0.734
AST	22.9 ± 14.2 U/L	22.3 ± 16.0 U/L	0.636
NEU	5.0 ± 2.1 × 10^9^/L	4.9 ± 2.4 × 10^9^/L	0.909
NEU%	0.6 ± 0.1 %	0.6 ± 0.1 %	0.085
MONO	0.6 ± 0.2 × 10^9^/L	0.6 ± 0.2 × 10^9^/L	0.036
MONO%	0.1 ± 0.0 %	0.1 ± 0.0 %	**0.034**
BASO	0.0 ± 0.0 × 10^9^/L	0.0 ± 0.0 × 10^9^/L	0.997
BASO%	0.0 ± 0.0 %	0.0 ± 0.0 %	0.718
EOS	0.1 ± 0.1 × 10^9^/L	0.2 ± 0.2 × 10^9^/L	0.062
EO%	0.0 ± 0.0 %	0.0 ± 0.0 %	**0.049**
UREA	5.6 ± 1.7 mmol/L	5.9 ± 1.8 mmol/L	**0.023**
UA	328.4 ± 98.2 μmol/L	335.2 ± 119.2 μmol/L	0.485
TBiL	10.8 ± 7.0 μmol/L	10.4 ± 5.2 μmol/L	0.448
TP	66.1 ± 5.6 g/L	65.0 ± 5.9 g/L	**0.019**
CL	106.8 ± 2.7 mmol/L	107.1 ± 2.8 mmol/L	0.298
LYM	2.0 ± 0.8 × 10^9^/L	2.1 ± 0.8 × 10^9^/L	0.314
LYM%	0.3 ± 0.1 %	0.3 ± 0.1 %	0.306
GLO	25.3 ± 4.1 g/L	25.2 ± 4.2 g/L	0.766
WBC	7.8 ± 2.4 × 10^9^/L	7.9 ± 2.6 × 10^9^/L	0.591
ALB	40.9 ± 3.6 g/L	39.8 ± 3.6 g/L	**< 0.001**
A/G	1.7 ± 0.3	1.6 ± 0.3	0.271
DBiL	3.3 ± 1.7 μmol/L	3.2 ± 1.8 μmol/L	0.799
RBC	4.7 ± 0.6 × 10^12^/L	4.7 ± 0.7 × 10^12^/L	0.479
HCT	0.4 ± 0.0 L/L	0.4 ± 0.0 L/L	0.067
MCV	88.4 ± 9.0 fL	88.1 ± 9.8 fL	0.717
MCHC	325.5 ± 13.1 g/L	323.2 ± 11.6 g/L	**0.034**
MCH	28.8 ± 3.5 pg	28.5 ± 3.8 pg	0.361
CREA	74.5 ± 18.5 μmol/L	78.4 ± 28.3 μmol/L	0.076
CysC	1.1 ± 0.3 mg/L	1.2 ± 0.4 mg/L	**0.003**
PLT	247.9 ± 68.1 × 10^9^/L	255.6 ± 67.4 × 10^9^/L	0.186
HGB	135.0 ± 17.0 g/L	131.7 ± 15.2 g/L	**0.02**
Ca	2.3 ± 0.1 mmol/L	2.2 ± 0.1 mmol/L	**0.012**
Na	141.1 ± 2.1 mmol/L	141.1 ± 2.1 mmol/L	1
K	4.0 ± 0.4 mmol/L	4.0 ± 0.4 mmol/L	0.821
IBil	7.3 ± 4.0 μmol/L	7.2 ± 3.8 μmol/L	0.61
DB/TB	0.3 ± 0.1	0.3 ± 0.1	0.94

Bold values indicate statistical significance (*P* < 0.05).

Furthermore, significant differences were observed in hemoglobin (HGB) (*P* = 0.020) and calcium (Ca) (*P* = 0.012), with higher HGB and Ca levels in the good prognosis group. No significant differences were found in other laboratory markers, such as creatinine (CREA), platelets (PLT), sodium (Na), and potassium (K).

Table 3 shows the differences in imaging measurements between the two groups. The DHI was significantly lower in the poor prognosis group (0.280) compared to the good prognosis group (0.302), with a statistically significant difference. Additionally, the average foraminal height in the poor prognosis group was 17.5 mm, which was significantly lower than the 18.0 mm in the good prognosis group. A significant statistical difference was also observed. Patients in the poor prognosis group had higher Modic changes and Pfirrmann grades, indicating more severe disc degeneration. There were no significant differences in FO and FT between the two groups. For LLA and SS, the poor prognosis group had significantly higher values, with an average of 39.3 for LLA and 34.9 for SS, compared to the good prognosis group.

**Table 3:** Imaging measurements comparison between good and poor prognosis groups.

Variable	Type	Good prognosis	Poor prognosis	*P*-value
		(*N* = 473)	(*N* = 192)	
DHI	Mean ± SD	0.302 ± 0.1	0.280 ± 0.1	**< 0.001**
Foraminal height	Mean ± SD	18.0 ± 2.2	17.5 ± 2.3	**0.014**
Modic	Level 0	304 (64.3%)	101 (52.6%)	**< 0.001**
	Level 1	120 (25.4%)	45 (23.4%)	
	Level 2	43 (9.1%)	38 (19.8%)	
	Level 3	6 (1.3%)	8 (4.2%)	
Pfirrmann	I	5 (1.1%)	0 (0%)	**< 0.001**
	II	125 (26.4%)	25 (13%)	
	III	322 (68.1%)	141 (73.4%)	
	IV	21 (4.4%)	24 (12.5%)	
	V	0 (0%)	2 (1%)	
FO	Mean ± SD	46.7 ± 9.3	45.8 ± 10.1	0.259
FT	Mean ± SD	5.9 ± 5.0	6.4 ± 5.1	0.29
LLA	Mean ± SD	36.3 ± 11.4	39.3 ± 9.3	**< 0.001**
SS	Mean ± SD	33.0 ± 8.9	34.9 ± 7.1	**0.004**

Bold values indicate statistical significance (*P* < 0.05).

As shown in Table 4, baseline VAS and ODI scores were comparable between the good- and poor-prognosis groups. Baseline VAS scores were 7.9 ± 0.8 and 8.0 ± 0.8, respectively (*P* = 0.582), while baseline ODI scores were 0.6 ± 0.1 and 0.6 ± 0.1, respectively (*P* = 0.202). Because the 12-month outcome groups were defined directly using postoperative VAS and ODI thresholds, inferential comparisons of 12-month VAS and ODI between the two groups were not performed to avoid circular interpretation.

**Table 4:** Baseline VAS and ODI scores according to 12-month outcome group.

Variable	Type	Good prognosis (*N* = 473)	Poor prognosis (*N* = 192)	*P*-value
Baseline VAS	Mean ± SD	7.9 ± 0.8	8.0 ± 0.8	0.582
Baseline ODI	Mean ± SD	0.6 ± 0.1	0.6 ± 0.1	0.202

#### 3.1.2 Clinical/conventional imaging model development

We first developed a structured clinical/conventional-imaging reference model and compared different algorithms within this feature domain. Table 5 presents the accuracy, recall, precision, F1 score, and AUC for different models in the training and validation cohorts. Figure 2A provides a graphical visualization of these model-performance metrics. Among the candidate algorithms, XGBoost achieved the highest accuracy in the internal validation cohort and was selected for subsequent model development. Figure 2B displays the top five variables ranked by XGBoost, which are Pfirrmann grade, Age, SS, DHI, and Modic grade.

**Table 5:** Performance comparison of candidate machine-learning models in the training and internal validation cohorts.

Model	Accuracy (Training cohort)	Accuracy (Test cohort)	Recall	Precision	Jaccard coef	F1	AUC
LR	0.679	0.579	0.605	0.359	0.291	0.451	0.716
SVM	0.444	0.398	0.711	0.281	0.252	0.403	0.453
LightGBM	0.769	0.759	0.184	0.875	0.179	0.304	0.761
BaggingClassifier	0.968	0.789	0.474	0.692	0.391	0.563	0.732
RF	0.88	0.789	0.474	0.692	0.391	0.563	0.706
XGBoost	0.951	0.805	0.526	0.714	0.435	0.606	0.725

**Figure 2:**
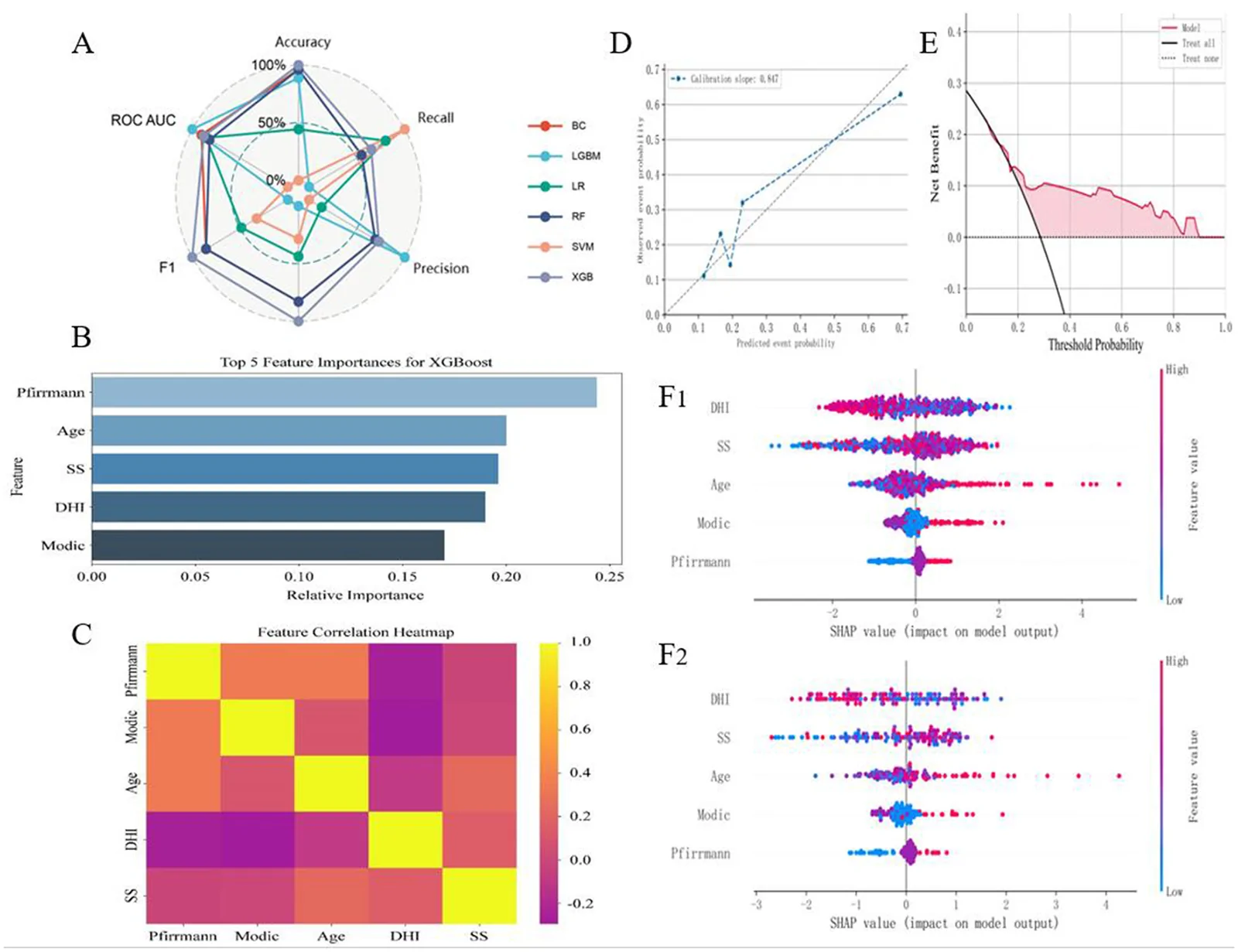
Development and interpretation of the structured clinical/conventional-imaging model. **(A)** Performance comparison of candidate machine-learning models. **(B)** Top five variables ranked by XGBoost feature importance. **(C)** Correlation heatmap of the five selected variables. **(D)** Calibration curve in the internal validation cohort. **(E)** Decision curve analysis in the internal validation cohort. **(F1, F2)** SHAP summary plots for the training and internal validation cohorts, respectively. Additional SHAP visualizations are provided in Supplementary Figure S2.

Figure 2C shows the correlation heatmap of the five important variables, where the depth of color is proportional to the correlation strength between features. To further assess the clinical utility of the XGBoost model in the validation set, calibration curves (Figure 2D) and decision curve analysis (Figure 2E) based on the validation cohort were plotted. The calibration slope was 0.847, indicating some deviation from ideal calibration; the corresponding decision curve analysis is shown in Figure 2E.

We also generated SHAP summary plots to characterize feature importance and the direction of feature effects in the structured clinical/conventional-imaging model. Figures 2F1, F2 show the SHAP value distributions in the training and internal validation cohorts, respectively, with features arranged according to their mean absolute SHAP values. DHI, SS, age, Modic grade, and Pfirrmann grade were the dominant contributors to model predictions. Additional SHAP visualizations, including feature-distribution heatmaps and representative single-patient waterfall plots, are provided in Supplementary Figure S2.

### 3.2 Radiomics and deep learning

#### 3.2.1 Radiomics

A total of 660 radiomic features were initially extracted from each imaging modality, yielding 1,320 radiomic features before feature selection. Following feature selection exclusively within the training cohort, five CT and five MRI radiomic features were retained. The selected features and their training-derived importance rankings are summarized in Table 6 and Figures 3A1, A2. Inter-reader reproducibility analysis demonstrated high agreement between the two senior spine surgeons. The mean Dice coefficients were 0.991 ± 0.004 for both CT and MRI ROI segmentation, with corresponding IoU values of 0.982 ± 0.007 and 0.981 ± 0.008, respectively. The ICC(A,1) values for ROI width, height, and area were 0.999, 0.999, and 0.999 for CT, and 0.997, 0.998, and 0.998 for MRI, respectively, indicating excellent inter-reader reliability. Detailed reproducibility results are provided in Supplementary Table S1.

**Table 6:** CT and MRI radiomics features selected from the training cohort.

CT radiomics features	MRI radiomics features
wavelet-L_glrlm _GrayLevelNonUniformityNormalized	wavelet-L_gldm _LowGrayLevelEmphasis
wavelet-L_ngtdm_Contrast	original_shape2D_MinorAxisLength
original_glrlm _GrayLevelNonUniformityNormalized	log-sigma-1-mm-3D_glrlm _LongRunHighGrayLevelEmphasis
lbp-2D_glszm _ZoneEntropy	log-sigma-1-mm-3D_gldm _DependenceNonUniformity
lbp-2D_glrlm _RunEntropy	log-sigma-1-mm-3D_gldm _SmallDependenceLowGrayLevelEmphasis

The five highest-ranking features from each radiomics domain were selected using XGBoost-based feature importance calculated exclusively within the training cohort. The selected features were subsequently used in the corresponding isolated radiomics models and the final multimodal model.

**Figure 3:**
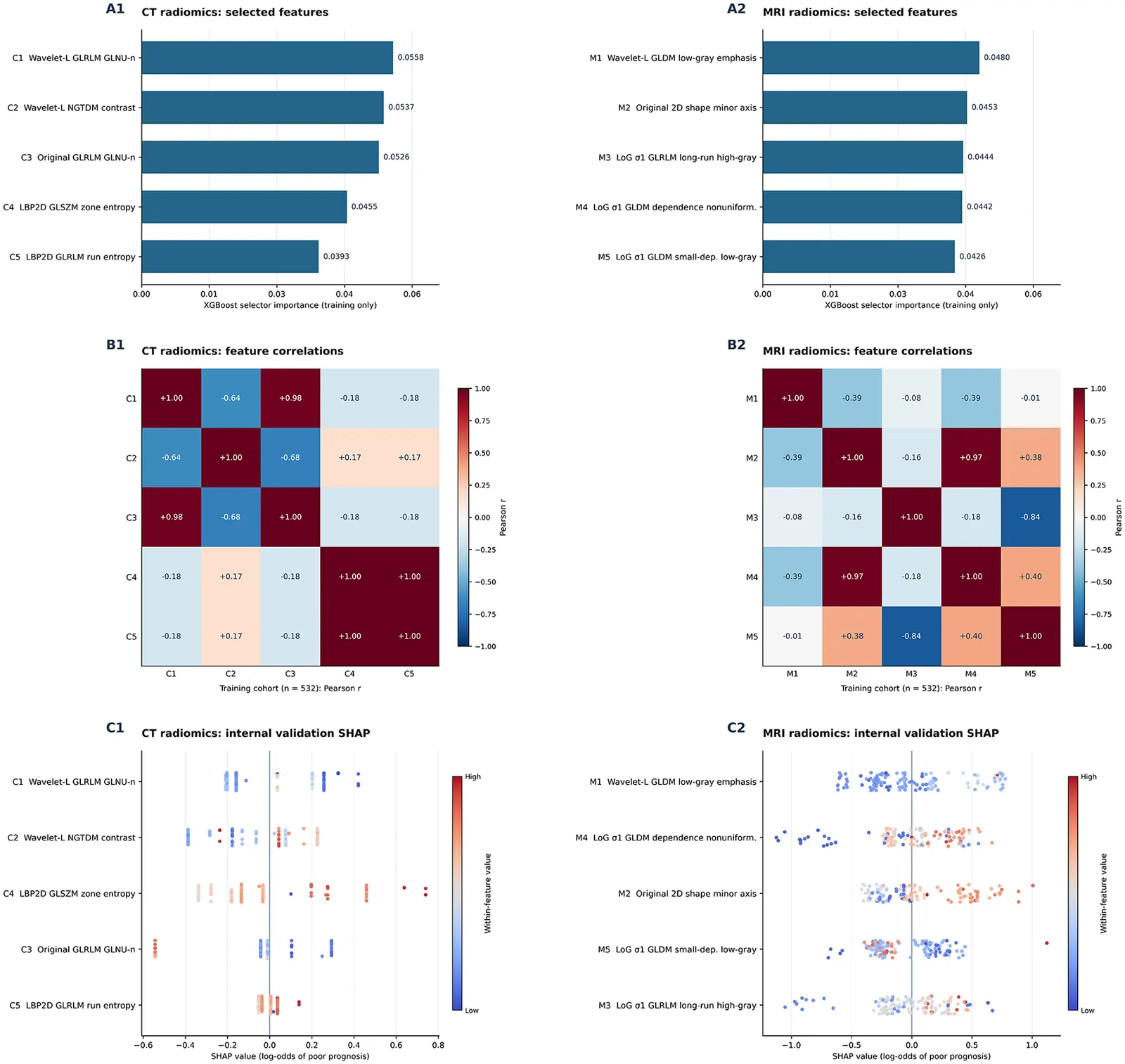
Training-derived CT and MRI radiomics feature selection and model interpretation. **(A1, A2)** Importance rankings of the five CT and five MRI radiomic features selected exclusively from the training cohort. The feature-importance values were calculated using the training-fitted XGBoost feature selector. **(B1, B2)** Pearson correlation matrices of the selected CT and MRI radiomic features in the training cohort (*n* = 532). **(C1, C2)** SHAP summary plots illustrating the contributions of the selected radiomic features to predictions made by the corresponding CT and MRI radiomics models in the internal validation cohort (*n* = 133). The identifiers C1–C5 and M1–M5 within the plots denote individual CT and MRI radiomic features, respectively, and correspond to the features listed in Table 6. SHAP values are expressed on the log-odds scale for the positive class (poor prognosis), and point colors indicate the relative observed values of each feature.

Correlation analysis of the selected features was performed in the training cohort (Figures 3B1, B2). Several radiomic features demonstrated strong correlations, particularly between the selected CT gray-level run-length features and between two selected MRI features. These findings indicate that some of the retained features contain overlapping information and should not be interpreted as independent predictors.

In the internal validation cohort, the isolated CT and MRI radiomics models achieved ROC-AUC values of 0.608 (95% CI, 0.509–0.707) and 0.632 (95% CI, 0.523–0.740), respectively. In the prospective validation cohort, the corresponding ROC-AUC values were 0.480 (95% CI, 0.358–0.602) and 0.498 (95% CI, 0.381–0.616), respectively.

SHAP analysis of the final modality-specific models in the internal validation cohort is presented in Figures 3C1, C2. The isolated radiomics models demonstrated limited discrimination, particularly in the prospective validation cohort.

#### 3.2.2 Deep-learning features

Previously generated deep-learning feature matrices based on a reported ViT-Base architecture were available for both CT and MRI. Each imaging modality contributed 768 deep-learning features per patient before feature selection. Following feature selection exclusively within the training cohort, five CT and five MRI deep-learning features were retained for the final modeling pipeline. The selected features and their training-derived importance rankings are summarized in Table 7 and Figures 4A1, A2, respectively.

**Table 7:** CT and MRI deep-learning features selected from the training cohort.

CT deep-learning features	MRI deep-learning features
CT_AX_Soft_feature_408	MRI_AX_T2_feature_503
CT_AX_Soft_feature_548	MRI_AX_T2_feature_533
CT_AX_Soft_feature_310	MRI_AX_T2_feature_166
CT_AX_Soft_feature_56	MRI_AX_T2_feature_449
CT_AX_Soft_feature_682	MRI_AX_T2_feature_461

Features were ranked according to XGBoost-based feature importance calculated exclusively within the training cohort. The same selected features were used in the corresponding isolated deep-learning models and the final multimodal model.

**Figure 4:**
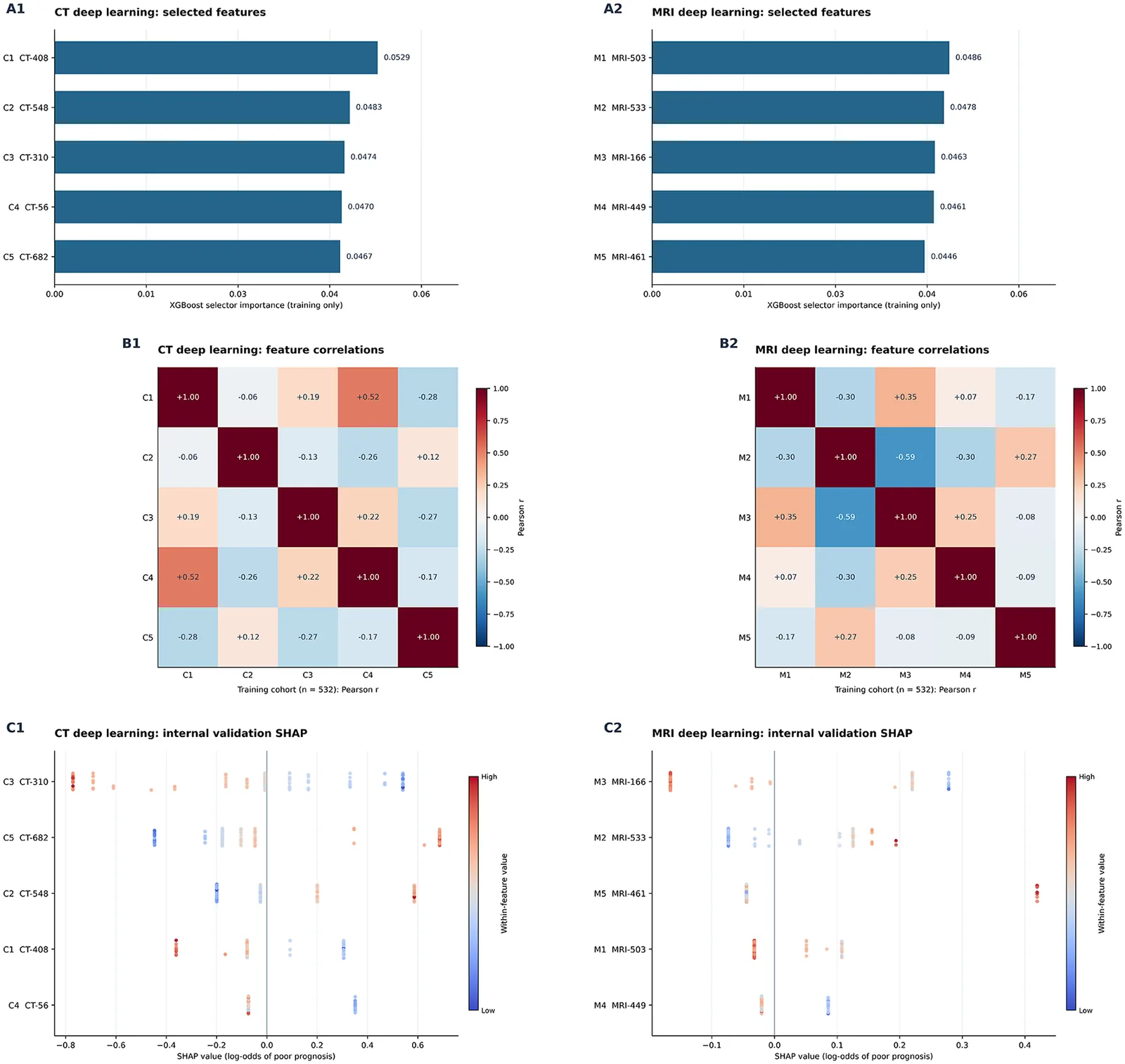
Training-derived CT and MRI deep-learning feature selection and model interpretation. **(A1, A2)** Importance rankings of the five CT and five MRI deep-learning features selected exclusively from the training cohort. The feature-importance values were calculated using the training-fitted XGBoost feature selector. **(B1, B2)** Pearson correlation matrices of the selected CT and MRI deep-learning features in the training cohort (*n* = 532). **(C1, C2)** SHAP summary plots illustrating the contributions of the selected deep-learning features to predictions made by the corresponding CT and MRI deep-learning models in the internal validation cohort (*n* = 133). The identifiers C1–C5 and M1–M5 within the plots denote individual CT and MRI deep-learning features, respectively, and correspond to the features listed in Table 7. SHAP values are expressed on the log-odds scale for the positive class (poor prognosis). These SHAP plots explain the downstream XGBoost predictions rather than the spatial attention of the original ViT network.

Correlation analysis of the selected deep-learning features was performed in the training cohort (*n* = 532), and the corresponding correlation matrices are presented in Figures 4B1, B2. SHAP analysis was subsequently performed to characterize the contributions of the selected features to predictions generated by the corresponding CT and MRI deep-learning models in the internal validation cohort (*n* = 133; Figures 4C1, C2). These SHAP analyses explain the predictions of the downstream XGBoost models rather than the spatial attention mechanisms of the original ViT network.

In the internal validation cohort, the isolated CT and MRI deep-learning models achieved ROC-AUC values of 0.765 (95% CI, 0.680–0.851) and 0.644 (95% CI, 0.542–0.746), respectively. In the prospective validation cohort, the corresponding ROC-AUC values were 0.515 (95% CI, 0.398–0.632) and 0.635 (95% CI, 0.521–0.748), respectively. The isolated deep-learning models demonstrated variable discrimination across the validation cohorts, with the CT deep-learning model showing reduced discrimination in the prospective cohort compared with the internal validation cohort.

The discriminatory performance of the four isolated imaging-based models was compared between the internal and prospective validation cohorts (Figure 5). The ROC-AUC values and corresponding 95% confidence intervals for all four isolated imaging-based models are presented separately for the internal validation cohort (Figure 5A) and the prospective validation cohort (Figure 5B). The isolated CT radiomics, MRI radiomics, and CT deep-learning models showed ROC-AUC values close to 0.50 in the prospective validation cohort.

**Figure 5:**
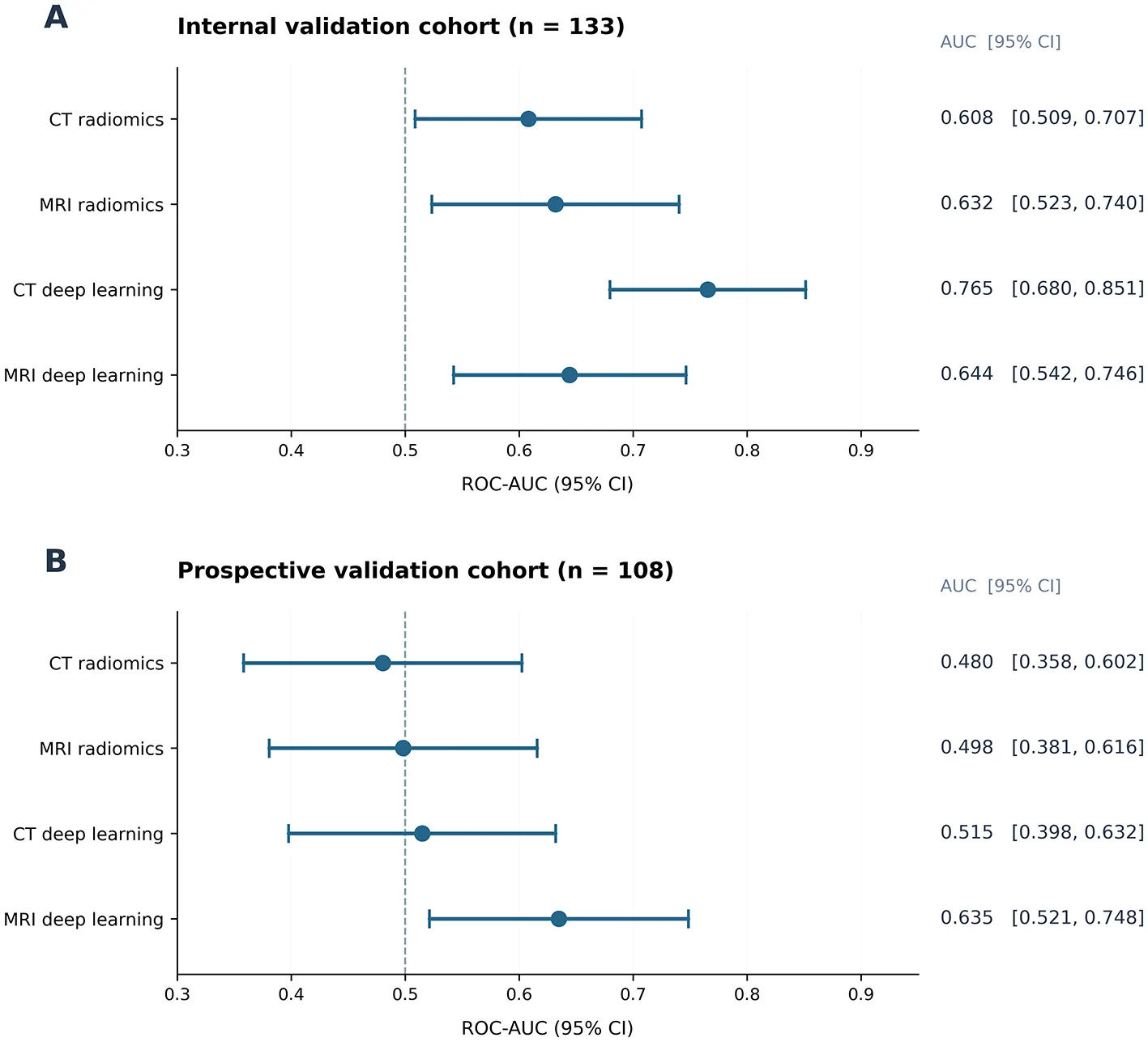
Discriminative performance of the isolated imaging-based models in the internal and prospective validation cohorts. **(A)** ROC-AUC values and corresponding 95% confidence intervals for the CT radiomics, MRI radiomics, CT deep-learning, and MRI deep-learning models in the internal validation cohort (*n* = 133). **(B)** Corresponding ROC-AUC values and 95% confidence intervals in the prospective validation cohort (*n* = 108). The points represent ROC-AUC estimates, and the horizontal error bars indicate their 95% confidence intervals. The dashed vertical line indicates an ROC-AUC of 0.50. All four models were fitted using the retrospective training cohort and subsequently evaluated without further downstream model fitting in either validation cohort.

### 3.3 Multimodal model and internal validation

A multimodal model was developed by integrating five feature domains: structured clinical and conventional imaging variables, CT radiomics features, MRI radiomics features, CT deep-learning features, and MRI deep-learning features. Following training-only feature selection, the five highest-ranking features from each domain were retained, resulting in a 25-feature multimodal representation.

In the internal validation cohort, the multimodal model achieved an ROC-AUC of 0.937 (95% CI, 0.900–0.975) and a PR-AUC of 0.849. The corresponding ROC-AUC values for the structured clinical/conventional-imaging, CT radiomics, MRI radiomics, CT deep-learning, and MRI deep-learning models were 0.939, 0.608, 0.632, 0.765, and 0.644, respectively. The multimodal model demonstrated discrimination comparable to that of the structured clinical/conventional-imaging model (△AUC = −0.002, *P* = 0.902, DeLong test), whereas it showed significantly better discrimination than each of the isolated radiomics and deep-learning models (all *P* < 0.001). The ROC and precision-recall curves are shown in Figures 6A, B, respectively.

**Figure 6:**
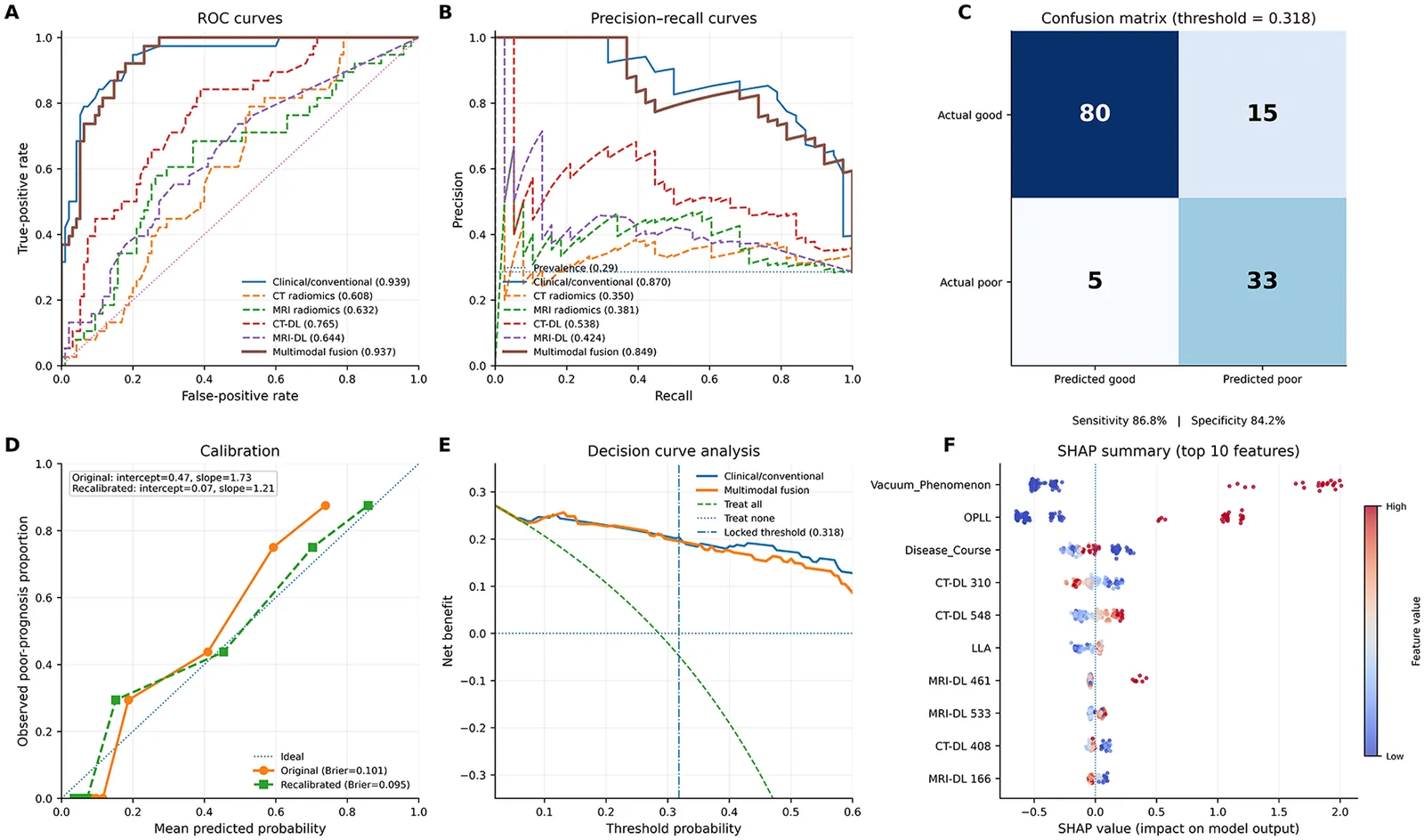
Internal validation of the multimodal model. **(A)** ROC curves for the structured clinical/conventional-imaging, CT radiomics, MRI radiomics, CT deep-learning, MRI deep-learning, and multimodal models. **(B)** Precision-recall curves for the same models. **(C)** Confusion matrix for the multimodal model at the locked training-derived threshold of 0.318. **(D)** Original and training-derived recalibrated calibration curves. **(E)** Decision curve analysis. **(F)** SHAP summary plot showing the ten most influential features in the internal validation cohort.

At the locked training-derived classification threshold of 0.318, the internal validation confusion matrix consisted of 80 true negatives, 15 false positives, 5 false negatives, and 33 true positives. The resulting sensitivity was 86.8%, specificity was 84.2%, PPV was 68.8%, NPV was 94.1%, F1 score was 0.767, balanced accuracy was 0.855, and overall accuracy was 85.0%. Detailed performance estimates and corresponding 95% confidence intervals are provided in Supplementary Table S2. The corresponding locked-threshold confusion matrix is shown in Figure 6C. Calibration analysis yielded a Brier score of 0.101, a calibration intercept of 0.473, and a calibration slope of 1.725. Training-derived logistic recalibration improved the Brier score to 0.095, with the calibration intercept and slope changing to 0.065 and 1.210, respectively. The original and training-derived recalibrated calibration curves are shown in Figure 6D.

These findings indicate that the multimodal model maintained strong discrimination in the internal validation cohort. However, its discrimination was comparable to that of the structured clinical/conventional-imaging model, suggesting that the added radiomics and deep-learning features provided complementary information rather than a substantial incremental gain in overall discrimination. Decision curve analysis and SHAP interpretation are presented in Figures 6E, F, respectively.

### 3.4 Prospective validation

A total of 108 prospectively recruited patients with complete 12-month outcome data were included in the final prospective validation analysis. Of these, 74 patients had good prognosis and 34 had poor prognosis. The prospective cohort was evaluated using the previously locked model without any modification of model parameters or decision threshold. The baseline demographic and clinical characteristics of the prospective validation cohort are summarized in Tables 8–10. Baseline VAS and ODI scores in the prospective validation cohort are summarized in Table 11. As shown in Table 8, the average age of the poor prognosis group (45.3 ± 16.7) was significantly higher than that of the good prognosis group (38.2 ± 10.8). In Table 10, the DHI was significantly lower in the poor prognosis group compared to the good prognosis group, and the Pfirrmann grade was higher in the poor prognosis group, with statistically significant differences. Baseline VAS and ODI scores were comparable between outcome groups in the prospective cohort (Table 11). Postoperative 12-month VAS and ODI were not compared inferentially between the groups because these variables constituted the outcome definition itself.

**Table 8:** Baseline Demographic and clinical characteristics of patients in the prospective validation cohort.

Variable	Type	Good prognosis (*N* = 74)	Poor prognosis (*N* = 34)	*P*-value
Gender	Female	19 (25.7%)	10 (29.4%)	0.863
	Male	55 (74.3%)	24 (70.6%)	
Age	Mean ± SD	38.2 ± 10.8	45.3 ± 16.7	**0.027**
BMI	Mean ± SD	23.8 ± 2.7	23.6 ± 3.4	0.724
Smoking	No	50 (67.6%)	22 (64.7%)	0.942
	Yes	24 (32.4%)	12 (35.3%)	
Hypertension	No	71 (95.9%)	31 (93.9%)	1
	Yes	3 (4.1%)	2 (6.1%)	
Diabetes	No	72 (97.3%)	31 (91.2%)	0.361
	Yes	2 (2.7%)	3 (8.8%)	
Disease duration	Mean ± SD	18.1 ± 27.2	22.0 ± 30.3	0.511
L3/4	No	73 (98.6%)	33 (97.1%)	1
	Yes	1 (1.4%)	1 (2.9%)	
L4/5	No	33 (44.6%)	13 (38.2%)	0.681
	Yes	41 (55.4%)	21 (61.8%)	
L5/S1	No	42 (56.8%)	22 (64.7%)	0.569
	Yes	32 (43.2%)	12 (35.3%)	
Surgical approach	Interlaminar approach	51 (68.9%)	24 (70.6%)	1
	Transforaminal approach	23 (31.1%)	10 (29.4%)	
Affected side	Right	22 (29.7%)	16 (47.1%)	0.125
	Left	52 (70.3%)	18 (52.9%)	
Herniation types	Protrusion	1 (1.4%)	0 (0%)	0.914
	Herniation	35 (47.3%)	17 (50.0%)	
	Extrusion	33 (44.6%)	15 (44.1%)	
	Sequestration	5 (6.8%)	2 (5.9%)	
Herniation location	Lateral type	65 (87.8%)	26 (76.5%)	0.296
	Central type	6 (8.1%)	6 (17.6%)	
	Far lateral type	3 (4.1%)	2 (5.9%)	

Bold values indicate statistical significance (*P* < 0.05).

**Table 9:** Laboratory test results in the prospective validation cohort.

Variable	Good prognosis (*N* = 74)	Poor prognosis (*N* = 34)	*P*-value
GGT	42.3 ± 38.2	43.1 ± 25.9	0.896
ALT	32.6 ± 23.1	32.1 ± 22.8	0.906
AST	25.8 ± 27.9	22.8 ± 10.5	0.424
NEU	4.9 ± 2.0	4.7 ± 2.0	0.523
NEU%	0.6 ± 0.1	0.6 ± 0.1	0.289
MONO	0.5 ± 0.2	0.5 ± 0.2	0.949
MONO%	0.1 ± 0.0	0.1 ± 0.0	0.843
BASO	0.0 ± 0.0	0.0 ± 0.0	0.562
BASO%	0.0 ± 0.0	0.0 ± 0.0	0.431
EOS	0.1 ± 0.1	0.2 ± 0.2	0.159
EO%	0.0 ± 0.0	0.0 ± 0.0	0.177
UREA	4.9 ± 1.4	4.8 ± 1.4	0.853
UA	343.7 ± 114.9	366.7 ± 92.8	0.309
TBiL	10.3 ± 4.7	8.8 ± 3.1	0.055
TP	74.6 ± 5.3	73.9 ± 4.5	0.494
CL	105.2 ± 12.0	104.0 ± 2.5	0.394
LYM	1.9 ± 0.6	2.1 ± 0.8	0.415
LYM%	0.3 ± 0.1	0.3 ± 0.1	0.391
GLO	30.6 ± 4.4	30.0 ± 4.6	0.514
WBC	7.5 ± 2.0	7.5 ± 2.1	0.818
ALB	44.0 ± 3.3	43.9 ± 2.8	0.853
A/G	1.5 ± 0.2	1.5 ± 0.3	0.443
DBiL	3.1 ± 1.6	2.7 ± 0.8	0.111
RBC	5.0 ± 0.6	5.0 ± 0.7	0.699
HCT	0.4 ± 0.1	0.4 ± 0.1	0.571
MCV	86.9 ± 8.5	87.0 ± 7.8	0.942
MCHC	333.5 ± 12.2	332.9 ± 10.6	0.810
MCH	29.1 ± 3.5	29.0 ± 3.1	0.958
CREA	71.3 ± 14.6	69.5 ± 15.6	0.568
CysC	0.7 ± 0.1	0.8 ± 0.2	0.067
PLT	272.1 ± 59.9	273.3 ± 68.5	0.924
HGB	144.3 ± 15.4	142.9 ± 18.8	0.682
Ca	2.6 ± 2.5	2.4 ± 0.1	0.377
Na	138.1 ± 11.5	139.2 ± 2.8	0.467
K	4.0 ± 0.3	4.1 ± 0.3	0.183
IBil	7.1 ± 3.4	6.0 ± 2.7	0.100
DB/TB	0.3 ± 0.1	0.3 ± 0.1	0.246

**Table 10:** Imaging measurements in the prospective validation cohort.

Variable	Type	Good prognosis	Poor prognosis	*P*-value
		(*N* = 74)	(*N* = 34)	
DHI	Mean ± SD	0.302 ± 0.041	0.283 ± 0.054	**0.039**
Foraminal height	Mean ± SD	16.6 ± 2.0	16.8 ± 2.2	0.661
Modic	Level 0	53 (71.6%)	20 (58.8%)	0.556
	Level 1	18 (24.3%)	11 (32.4%)	
	Level 2	2 (2.7%)	2 (5.9%)	
	Level 3	1 (1.4%)	1 (2.9%)	
Pfirrmann	I	1 (1.4%)	0 (0%)	**0.006**
	II	14 (18.9%)	0 (0%)	
	III	54 (73.0%)	26 (76.5%)	
	IV	5 (6.8%)	8 (23.5%)	
	V	0 (0%)	0 (0%)	
FO	Mean ± SD	47.5 ± 9.3	46.9 ± 10.9	0.767
FT	Mean ± SD	4.6 ± 4.1	5.3 ± 4.6	0.479
LLA	Mean ± SD	32.1 ± 11.2	34.5 ± 14.0	0.335
SS	Mean ± SD	30.4 ± 8.0	31.6 ± 10.2	0.486

Bold values indicate statistical significance (*P* < 0.05).

**Table 11:** Baseline VAS and ODI scores according to 12-month outcome group in the prospective validation cohort.

Variable	Type	Good prognosis	Poor prognosis	*P*-value
		(*N* = 74)	(*N* = 34)	
Baseline VAS	Mean ± SD	8.1 ± 0.8	8.1 ± 0.8	0.705
Baseline ODI	Mean ± SD	0.6 ± 0.1	0.6 ± 0.1	0.33

Poor prognosis was treated as the positive class during model evaluation. In the prospective validation cohort, the multimodal model achieved an ROC-AUC of 0.976 (95% CI, 0.951–1.000) and a PR-AUC of 0.941, indicating strong discrimination in a prospectively collected validation population. The ROC-AUC values for the structured clinical/conventional-imaging, CT radiomics, MRI radiomics, CT deep-learning, and MRI deep-learning models were 0.974, 0.480, 0.498, 0.515, and 0.635, respectively. The difference in ROC-AUC between the multimodal and structured clinical/conventional-imaging models was not statistically significant (△AUC = 0.002, *P* = 0.744, DeLong test), whereas the multimodal model significantly outperformed the isolated radiomics and deep-learning models (all *P* < 0.001). The ROC and precision-recall curves are shown in Figures 7A, B, respectively.

**Figure 7:**
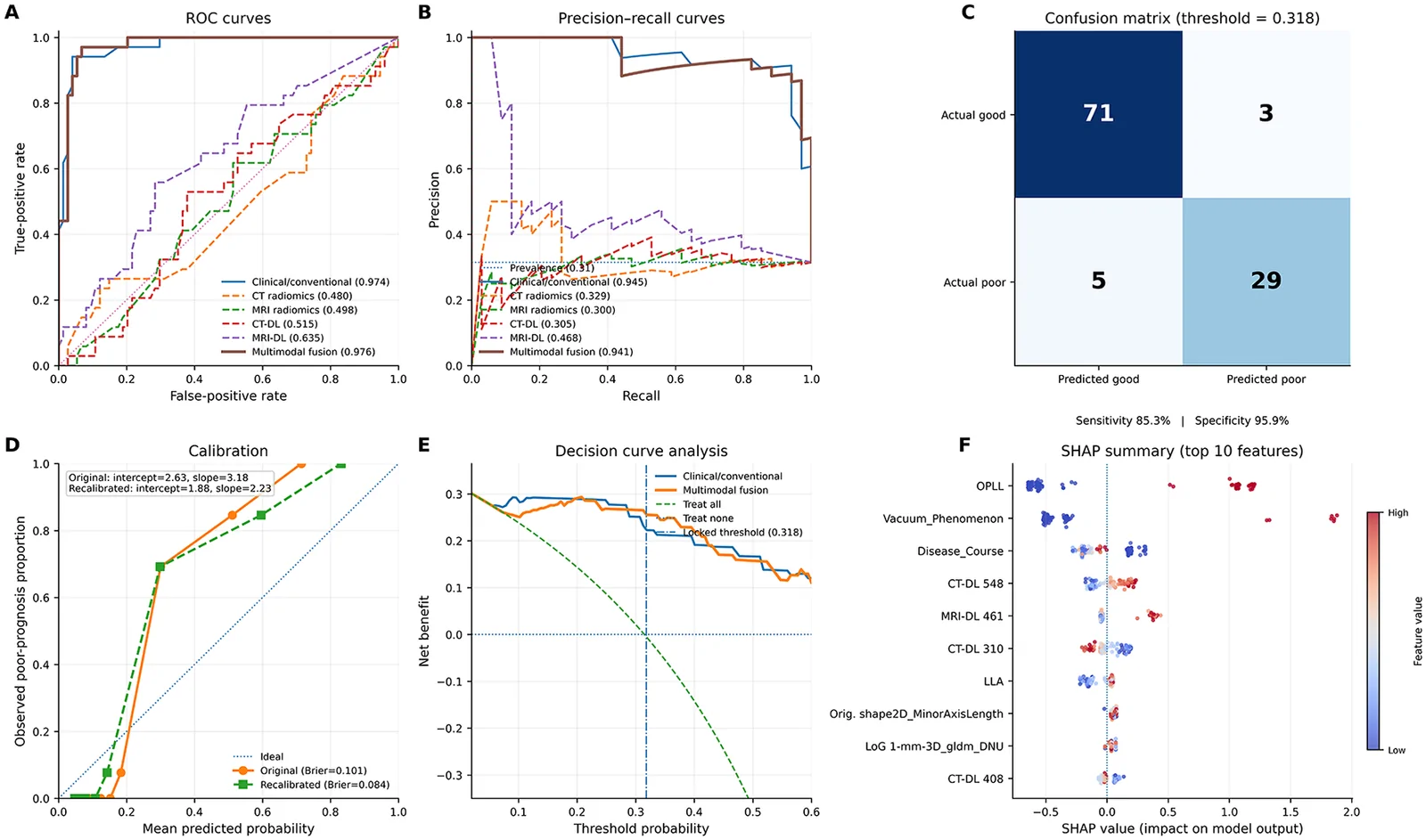
Prospective validation of the locked multimodal model. **(A)** ROC curves for the structured clinical/conventional-imaging, CT radiomics, MRI radiomics, CT deep-learning, MRI deep-learning, and multimodal models. **(B)** Precision-recall curves for the same models. **(C)** Confusion matrix for the locked multimodal model at the prespecified training-derived threshold of 0.318. **(D)** Original and training-derived recalibrated calibration curves. **(E)** Decision curve analysis. **(F)** SHAP summary plot showing the ten most influential features in the prospective validation cohort.

The influence of classification-threshold selection was further examined because the conventional probability threshold of 0.50 favored specificity at the expense of sensitivity. At a threshold of 0.50, the multimodal model yielded 72 true negatives, two false positives, 15 false negatives, and 19 true positives, corresponding to a sensitivity of 55.9% and a specificity of 97.3%.

When the prespecified training-derived threshold of 0.318 was applied without further optimization in the prospective cohort, the confusion matrix consisted of 71 true negatives, three false positives, 5 false negatives, and 29 true positives. Sensitivity was 85.3% (95% CI, 69.9–93.6%) and specificity was 95.9% (95% CI, 88.7–98.6%). PPV was 90.6%, NPV was 93.4%, F1 score was 0.879, balanced accuracy was 0.906, and overall accuracy was 92.6%. Detailed performance estimates and corresponding 95% confidence intervals are provided in Supplementary Table S2. The locked-threshold confusion matrix is shown in Figure 7C.

Calibration analysis demonstrated residual probability miscalibration despite strong discrimination. Before recalibration, the prospective cohort had a Brier score of 0.101, a calibration intercept of 2.629, and a calibration slope of 3.180. Logistic recalibration derived exclusively from out-of-fold predictions in the training cohort improved the Brier score to 0.084 and reduced the calibration intercept and slope to 1.877 and 2.231, respectively. Although recalibration improved probability calibration, residual miscalibration remained in the prospective cohort, supporting the need for further prospective validation and calibration assessment before using absolute predicted probabilities for clinical decision-making. The corresponding original and recalibrated calibration curves are shown in Figure 7D. Decision curve analysis and SHAP interpretation are presented in Figures 7E, F, respectively. The 95% confidence intervals for the Brier score, calibration intercept, and calibration slope before and after recalibration are reported in Supplementary Table S2.

## 4 Discussion

The present study developed a multimodal model integrating structured clinical and conventional imaging variables, CT and MRI radiomics features, and CT and MRI deep-learning features. The model achieved ROC-AUC values of 0.937 and 0.976 in the internal and prospective validation cohorts, respectively. Although the multimodal model demonstrated higher discrimination than the isolated radiomics and deep-learning models, its ROC-AUC was comparable to that of the structured clinical/conventional-imaging model in both validation cohorts. These findings indicate that the inclusion of quantitative imaging features did not produce a statistically significant incremental improvement in discrimination over the structured clinical/conventional-imaging model in the present study (Pedersen et al., 2022).

The potential clinical relevance of the present model lies in the preoperative identification of patients who may not achieve a minimal symptom state at 12 months after surgery. At the training-derived classification threshold of 0.318, the multimodal model achieved a sensitivity of 85.3% and a specificity of 95.9% in the prospective validation cohort. These findings suggest that the model may help identify patients who warrant closer postoperative monitoring. However, its high discriminative performance should not be interpreted as evidence of sufficiently accurate individual risk estimation (Riley et al., 2024a). The prospective validation cohort demonstrated residual probability miscalibration, indicating that predicted probabilities may not directly correspond to observed outcome frequencies. Therefore, additional calibration assessment and prospective evaluation of clinical utility are necessary before the model can be used to guide individualized treatment decisions (Van Calster et al., 2019).

Previous studies have investigated postoperative pain, disability, and imaging-related factors associated with recovery after lumbar discectomy (Berg et al., 2024). The present study extends this clinical context by evaluating several preoperative information domains within a common model-development and validation framework. Importantly, although the isolated imaging-based models generally demonstrated lower discrimination than the full multimodal model, the structured clinical/conventional-imaging model achieved a comparable ROC-AUC in both validation cohorts. These findings suggest that the potential contribution of complex imaging representations should be assessed against an appropriately developed clinical reference model rather than inferred solely from comparisons with isolated imaging-based models (Pedersen et al., 2022).

The final multimodal model incorporated five structured clinical and conventional imaging features selected exclusively from the training cohort: vacuum phenomenon, ossification of the posterior longitudinal ligament (OPLL), lumbar spondylolisthesis, disease course, and lumbar lordosis angle (LLA). These variables represent different aspects of preoperative clinical presentation and conventional imaging findings. However, their selection by the XGBoost-based procedure does not establish independent causal relationships with postoperative outcomes. Furthermore, the relative importance and direction of feature contributions may depend on interactions and correlations among the included variables. The SHAP analyses should therefore be interpreted as explanations of model predictions rather than evidence of underlying causal mechanisms (Ponce-Bobadilla et al., 2024).

Although radiomics and deep-learning features were retained during model development, the isolated imaging-based models demonstrated limited or variable discrimination across the validation cohorts. Furthermore, the structured clinical/conventional-imaging model achieved a ROC-AUC comparable to that of the full multimodal model. These findings indicate that the additional quantitative imaging representations did not yield a statistically significant incremental improvement in discrimination in the present cohorts. The SHAP analyses characterized the contributions of the selected features to downstream XGBoost predictions; however, the abstract deep-learning features cannot be directly attributed to specific anatomical structures or biological mechanisms without additional image-level validation. Moreover, strong correlations among certain selected radiomic features suggest potential redundancy rather than independent prognostic information.

In a supplementary ablation analysis, exclusion of the CT and MRI deep-learning features did not result in an observed reduction in ROC-AUC in either validation cohort. The 15-feature model achieved ROC-AUC values of 0.939 and 0.978 in the internal and prospective validation cohorts, respectively, compared with 0.937 and 0.976 for the full 25-feature model. The paired differences were not statistically significant in either cohort (*P* = 0.886 and *P* = 0.726, respectively). These results indicate that the observed discrimination of the full model was not dependent on the inclusion of the deep-learning feature domains in the analyzed cohorts. However, the findings do not establish statistical equivalence or noninferiority and do not resolve the incomplete provenance of the original ViT feature-extraction process.

Several limitations should be considered when interpreting the present findings. First, the prospective validation cohort included only 108 patients, of whom 34 experienced poor prognosis. This relatively small number of outcome events limits the precision of performance estimates, particularly for calibration and subgroup-specific analyses (Riley et al., 2024b). Despite the use of training-only feature selection, stratified cross-validation, and independent validation cohorts, overfitting cannot be completely excluded because high-dimensional radiomics and deep-learning features were evaluated relative to a limited number of outcome events. Second, the study was conducted within a geographically limited regional setting, and the prospective validation cohort was derived from a single center, which may restrict generalizability to other institutions and populations. Third, model performance was dependent on the selected classification threshold. In the prospective validation cohort, use of the conventional threshold of 0.50 resulted in a sensitivity of 55.9%, whereas application of the prespecified training-derived threshold of 0.318 increased sensitivity to 85.3%. These findings indicate that the false-negative rate is sensitive to the operating threshold, and the optimal decision threshold requires further independent prospective evaluation before clinical implementation. Fourth, despite the high ROC-AUC observed in the prospective cohort, residual probability miscalibration remained, with a calibration intercept of 2.629 and a slope of 3.180 before recalibration. Although training-derived logistic recalibration improved the Brier score, it did not fully resolve the calibration discrepancies. Additional multicenter validation with larger samples and prespecified calibration assessment is therefore necessary before clinical implementation.

Several radiomics-related limitations should also be acknowledged. Although inter-reader agreement for ROI delineation was high between the two senior spine surgeons, repeated segmentation by the same reader was not available, and intra-reader reproducibility could therefore not be quantified. Furthermore, the present two-dimensional radiomics workflow did not include explicit voxel-spacing resampling, intensity normalization, or scanner-specific harmonization, and no independent phantom-based IBSI compliance verification was performed. These factors may affect the reproducibility and transportability of quantitative radiomic features across scanners, acquisition protocols, and institutions (Soliman et al., 2022). Future multicenter studies should incorporate standardized image-acquisition protocols, prospective inter- and intra-reader repeat segmentation, feature-level reproducibility assessment, and appropriate image harmonization procedures.

An additional limitation concerns the reproducibility and provenance of the deep-learning features. Although the original feature-extraction records identified the use of a ViT-Base architecture and the available feature matrices contained 768 features per imaging modality, the original model implementation and configuration were not retained. Consequently, the pretrained weight source, specific feature-output layer, input normalization parameters, and potential fine-tuning procedures could not be independently verified. Although downstream feature selection and XGBoost model development were restricted to the training cohort, the absence of the original ViT training records limited the reproducibility assessment of the upstream feature-extraction process. Accordingly, although the internal and prospective validation cohorts were excluded from downstream supervised feature selection and XGBoost model fitting, potential information leakage during the historical upstream ViT feature-generation process could not be independently excluded. In addition, because the original ViT model weights and implementation were unavailable, network-level interpretability analyses such as Grad-CAM or attention-map visualization could not be reliably generated retrospectively. Future studies should retain the complete network configuration, model weights, image-preprocessing procedures, and training records to facilitate independent replication and verification of the entire multimodal modeling pipeline (Collins et al., 2024a).

The primary outcome in this study was defined using an absolute 12-month minimal symptom state rather than MCID achievement. These approaches address different clinical questions: the former reflects residual symptom burden at a prespecified postoperative time point, whereas MCID quantifies the magnitude of change from baseline. Published MCID thresholds for VAS and ODI vary according to the population, procedure, anchor, and calculation method (Koehne et al., 2026). Therefore, the present outcome definition should not be interpreted as equivalent to an MCID-based definition, and future multicenter studies should evaluate the robustness of the model across alternative clinically meaningful outcome definitions, including MCID-based criteria.

Future investigations should prioritize multicenter validation in populations with different clinical characteristics and imaging-acquisition protocols. A fully documented and reproducible image-feature extraction pipeline, including explicit ViT architecture, pretrained weights, preprocessing procedures, and training strategy, should be established before further evaluation of the complete multimodal model. Subsequent studies should also assess feature-level radiomics reproducibility, calibration stability, and the incremental clinical value of quantitative imaging features beyond established clinical and conventional imaging variables. Prospective clinical-impact studies will ultimately be required to determine whether model-assisted prognostic assessment improves clinical decision-making or patient outcomes. Although the prospective cohort provided temporal validation of the locked model, it was derived from a single center and requires further evaluation in larger multicenter cohorts. In addition, the relatively limited number of outcome events in the prospective cohort may have contributed to uncertainty in calibration estimates and should be considered when interpreting the model performance. Future multicenter studies with prospectively collected data, standardized imaging protocols, and fully reproducible feature-extraction workflows are required to further validate the clinical utility and generalizability of the proposed model.

## 5 Conclusion

The multimodal prognostic model demonstrated strong discrimination for predicting 12-month outcomes after minimally invasive surgery for lumbar disc herniation in the analyzed internal and prospective validation cohorts. However, its ROC-AUC was comparable to that of the structured clinical/conventional-imaging model, indicating that the additional radiomics and deep-learning features did not provide a statistically significant incremental improvement in discrimination. The model also exhibited residual probability miscalibration in the prospective validation cohort. Furthermore, the incomplete provenance of the original ViT feature-extraction process limits independent verification of the complete multimodal modeling pipeline. Further multicenter validation, external recalibration where necessary, standardized image-feature extraction, and prospective clinical-impact evaluation are required before potential clinical implementation.

## Data Availability

The raw data supporting the conclusions of this article will be made available by the authors, without undue reservation.

## Supplementary material

Supplementary Figure S1 Measurement of Key Imaging Features in CT and MRI (e.g., Disc Height Index, Pfirrmann Grade, Modic Changes, Lumbar Lordosis Angle).

Supplementary Figure S2 Additional SHAP-based interpretation of the structured clinical/conventional-imaging model. **(A1, A2)** SHAP feature-distribution heatmaps in the training cohort and internal validation cohort, respectively. The heatmaps illustrate the distribution and direction of feature contributions across individual patients. **(B1, B2)** Representative single-patient SHAP waterfall plots from the training cohort and internal validation cohort, respectively, illustrating the contributions of individual features to the predicted model output.

Supplementary Table S1 Inter-reader reproducibility of CT and MRI ROI segmentation. Inter-reader reproducibility was evaluated in the 108 patients of the prospective validation cohort independently annotated by two senior spine surgeons. DSC and IoU are reported as mean ± standard deviation across patients. ICC(A,1) denotes a two-way random-effects, absolute-agreement, single-measure intraclass correlation coefficient. The 95% confidence intervals for ICC(A,1) were estimated by nonparametric bootstrap resampling (10,000 replicates; random seed = 42).

Supplementary Table S2 Performance of the locked multimodal model in the internal and prospective validation cohorts. Poor prognosis was treated as the positive class. All threshold-based metrics were calculated at the locked classification threshold of 0.318, derived exclusively from out-of-fold predictions in the training cohort. ROC-AUC confidence intervals are the DeLong confidence intervals reported in the main manuscript. Wilson score 95% confidence intervals were used for sensitivity, specificity, PPV, NPV, and overall accuracy. Stratified nonparametric bootstrap resampling (10,000 replicates; random seed = 42) was used for 95% confidence intervals of PR-AUC, F1 score, balanced accuracy, and Brier score. Calibration intercept and slope were estimated by logistic calibration of observed outcome on the logit of predicted probability; 95% confidence intervals are Wald intervals from the calibration model. Training-derived logistic recalibration was fitted using training data only and then applied unchanged to both validation cohorts.
